# Pollen exposure and hospitalization due to asthma exacerbations: daily time series in a European city

**DOI:** 10.1007/s00484-017-1369-2

**Published:** 2017-05-12

**Authors:** Nicholas J. Osborne, Ian Alcock, Benedict W. Wheeler, Shakoor Hajat, Christophe Sarran, Yolanda Clewlow, Rachel N. McInnes, Deborah Hemming, Mathew White, Sotiris Vardoulakis, Lora E. Fleming

**Affiliations:** 1 European Centre for Environment and Human Health, University of Exeter Medical School, Truro, Cornwall UK; 2 School of Public Health and Community Medicine, University of New South Wales, Sydney, NSW 2052 Australia; 3 London School of Hygiene and Tropical Medicine, London, UK; 4 Met Office Hadley Centre, Fitzroy Road, Exeter, EX1 3PB UK; 5 Environmental Change Department, Centre for Radiation, Chemical and Environmental Hazards, Public Health England, Chilton, Oxon, OX11 0RQ UK

**Keywords:** Pollen, Asthma, Air pollution, Grass pollen, Tree pollen, Time series

## Abstract

Exposure to pollen can contribute to increased hospital admissions for asthma exacerbation. This study applied an ecological time series analysis to examine associations between atmospheric concentrations of different pollen types and the risk of hospitalization for asthma in London from 2005 to 2011. The analysis examined short-term associations between daily pollen counts and hospital admissions in the presence of seasonal and long-term patterns, and allowed for time lags between exposure and admission. Models were adjusted for temperature, precipitation, humidity, day of week, and air pollutants. Analyses revealed an association between daily counts (continuous) of grass pollen and adult hospital admissions for asthma in London, with a 4–5-day lag. When grass pollen concentrations were categorized into Met Office pollen ‘alert’ levels, ‘very high’ days (vs. ‘low’) were associated with increased admissions 2–5 days later, peaking at an incidence rate ratio of 1.46 (95%, CI 1.20–1.78) at 3 days. Increased admissions were also associated with ‘high’ versus ‘low’ pollen days at a 3-day lag. Results from tree pollen models were inconclusive and likely to have been affected by the shorter pollen seasons and consequent limited number of observation days with higher tree pollen concentrations. Future reductions in asthma hospitalizations may be achieved by better understanding of environmental risks, informing improved alert systems and supporting patients to take preventive measures.

**Electronic supplementary material:**

The online version of this article (doi:10.1007/s00484-017-1369-2) contains supplementary material, which is available to authorized users.

## Introduction

Over the last 20 years, there has been an increase in the prevalence of asthma worldwide both in the high-income and in the less-industrialized world, placing an increased burden on health systems (Asher et al. 2006; WHO 2016). By 2006, across the 37 countries (high medium and low income on four continents) in the International Study of Asthma and Allergies in Childhood (ISAAC) study, the mean prevalence amongst 6–7 year olds of asthma was 12.6% and that of allergic rhinoconjunctivitis 8.5%. The total Global Burden of Disease due to asthma is estimated to be around 25 million disability-adjusted life years (DALYs) and 11 million years of life lost (YLLs) per year (WHO 2016).

Allergy and asthma both increased in prevalence in the UK population over a 20-year period (Anderson et al. 2004), with asthma prevalence appearing to have plateaued but allergy prevalence having continued to climb (Prescott and Allen 2011). UK figures in Phase 3 of the ISAAC study were amongst some of the highest, with a prevalence of 20.9% for asthma and 10.1% for allergic rhinoconjunctivitis amongst 6–7 year olds (Asher et al. 2006). Adult asthma prevalence in the UK has been measured at 17.6% (self-reported doctor diagnosed) and 18.2% (clinical asthma) (To et al. 2012). The direct costs of asthma to the UK National Health Service (NHS) are estimated at approximately £1 billion annually, with additional indirect costs (e.g. due to time off work and loss of productivity) of around £6 billion (NHS 2013). Although asthma attacks are usually controllable with treatment, a proportion of patients fail to manage their illness adequately. In the UK, such asthma exacerbations (an increase in severity of the disease and in the case of asthma and its signs and symptoms, e.g. shortness of breath) lead to 50,000 hospital admissions per annum, over 1200 deaths, and an annual spend of £800 million on pharmaceuticals (NHS 2013).

Factors that may induce allergic reactions and asthma exacerbations are wide-ranging and, depending on the individual triggers, include pollen, house dust mites, air pollution, weather changes, exercise, airborne irritants such as tobacco smoke, emotional factors, occupational sensitizers, and some pharmaceuticals and foods (SIGN and BTS 2011). The role of pollen in asthma exacerbation is increasingly understood (Osborne and Eggen 2015). For instance, a significant association between grass pollen exposure and hospital admission for asthma has been reported in Australia (in both adults (Erbas et al. 2007) and children (Erbas et al. 2012)), France (Huynh et al. 2010), UK (Lewis et al. 2000), Spain (Tobías et al. 2004; Altzibar et al. 2014), Italy (Ruffoni et al. 2013), Hungary (Makra et al. 2012), and the USA (Jariwala et al. 2014; Gleason et al. 2014; Darrow et al. 2012). Other health outcomes previously associated with acute pollen exposures include allergic rhinitis, food allergy (Datema et al. 2015), eosinophilic esophagitis (van Rhijn et al. 2013), cardiovascular events (Brunekreef et al. 2000), preterm births (Lavigne et al. 2014), and mental health impacts (Qin et al. 2013). There is evidence that the levels (Negrini et al. 2011; Ziello et al. 2012) and potentially the allergenicity of pollen are increasing with time, possibly associated with global climate change (Singer et al. 2005; Vardoulakis and Heaviside 2012).

Pollen concentrations are highly variable on a daily basis due to a number of factors including plant pollen production and weather conditions. Pollen may travel long distances, and the concentration of pollen in the air (particles per m^3^) is therefore not just a localized phenomenon. Measured pollen concentrations have been found to correlate across distances of 20 km (Erbas et al. 2007) and 41 km (Pashley et al. 2009), and the potential exists for pollen to travel much further, including trans-continentally (Skjoth et al. 2007). In the UK, pollen from tree species is released in the March to April period, and grass pollen (from numerous species) during the period mid-May to July in most years. Weed pollen is released from the end of June until September.

In addition to the types of source vegetation, factors that could be important in influencing pollen-asthma associations include precipitation, humidity, thunderstorms, wind, periods of dry weather, atmospheric blocking, and heat. These may well be difficult to separate as individual factors, as they are often strongly linked (e.g. temperature and pollination in plants), and it can be difficult to identify sufficiently large data sets to perform the appropriate stratified analyses. The role of thunderstorms/lightning in the increased risk of hospitalization due to pollen has also been recorded in various countries (Newson et al. 1998; Bellomo et al. 1992; D’Amato et al. 2005), but it is still unclear how often this occurs, nor its potential burden on the health system (Dabrera et al. 2013).

The potential exists for air pollution, primarily from traffic and other combustion activities (measured as PM_10_, PM_2.5_, NO_*x*_) and ground-level ozone, to interact with pollen, synergistically increasing the negative effects of pollen on respiratory health (Diaz-Sanchez et al. 1997). High levels of air pollution have been found to increase the allergenicity of pollen in plants growing in areas of high air pollution (Ghiani et al. 2012). However, some epidemiological evidence finds no effect from air pollution on the relationship between pollen and asthma exacerbation, so the interaction is not entirely clear (Lewis et al. 2000; Tobías et al. 2004).

The current study extends previous work by examining the relationship between daily pollen counts for a number of plant species/genus and hospitalizations for asthma. Reflecting the issue of growing urbanization, the current work focuses on the pollen-asthma relationship in a large urban conurbation. London was chosen as a study site as it has a large population with high density of population and health service provision, whilst having pollen concentrations (grains/m^3^) equivalent to other areas in the UK that have been monitored on a daily basis for a number of years. Allowing for lag periods and potential confounding, we hypothesized that days with higher pollen counts are associated with higher emergency hospital admissions for asthma.

## Materials and methods

We used an ecological time series analysis to examine associations between daily pollen concentrations and hospital admissions for asthma in London. This approach allows for the investigation of potential time lags between pollen exposure and hospitalization for asthma, since we do not necessarily expect admissions to occur on the same day as pollen exposure (Ito et al. 2015; Bhaskaran et al. 2013; Erbas et al. 2012). The approach also permits simultaneous consideration of the role of daily variation in meteorological conditions and air pollutant concentrations in predicting admission rates. The following sections describe the data sources regarding the primary health outcome, exposures of interest, and potential confounders included in analyses.

### Study site and population

Greater London is the UK’s largest city, and covers 1572 km^2^ with a population of approximately 8.2 million (2011 Census, https://www.ons.gov.uk/census). It has a temperate oceanic climate (Köppen-Geiger climate classification: Cfb—warm temperate, fully humid, warm summer (Kottek et al. 2006)), with average daily mean temperature (1980–2010) of 18.3 °C and 45 mm precipitation in July (Met Office 2017). London has 40% greenspace or open water by area, and over 2000 species of flowering plant have been recorded (LNHS 2017).

### Asthma outcome data

Counts of emergency in-patient admissions for asthma (ICD-10 J45 and J46) for all residents of Greater London (specifically those within the London Government Office Region) were derived for the observation days from Hospital Episode Statistics (HES) data for years 2005–2011 (linked with available pollen data as described below). HES captures all in-patient admissions to National Health Service hospitals in England, and whilst it is fundamentally an administrative database, it has been used extensively for similar epidemiological research (Atkinson et al. 2006). We restricted the sample to the working age population (16–64 years) to focus on adult asthma, excluding both children and older people (the latter would have a higher prevalence of comorbidities such as chronic obstructive pulmonary disease [COPD]).

### Pollen data

**Fig. 1:**
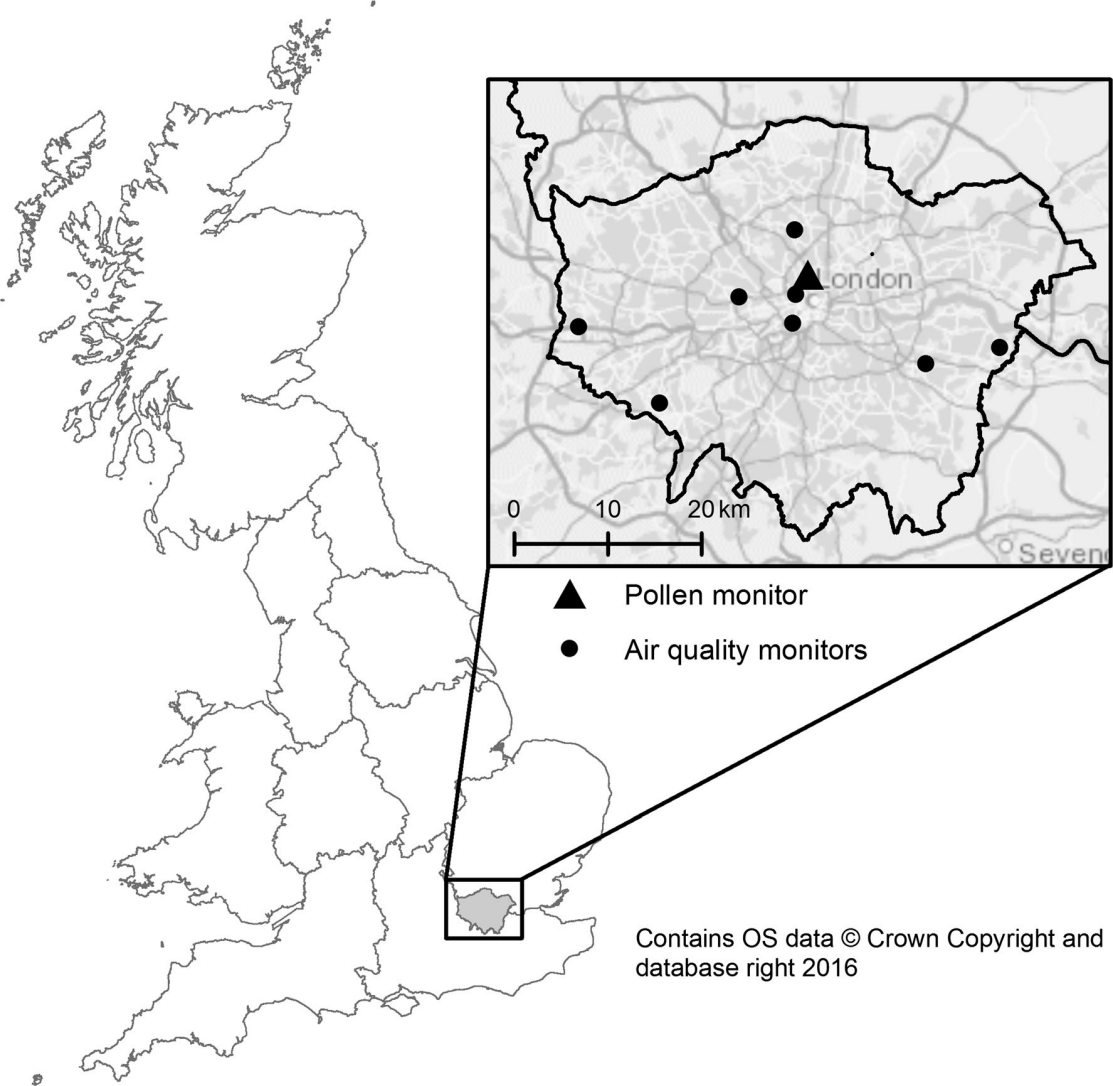
Locations of pollen and air quality monitors, within London Government Office Region

Airborne pollen was collected at a single pollen monitoring site in Islington, central London, from 2005 to 2011 (see Fig. 1) using a Burkard 7-day volumetric trap in accordance with British Aerobiology Federation protocols (BAF 1995). The drum was changed at 09:00 daily, and the tape prepared for microscopy. Pollen grains were identified to family (all species included), and counted by trained specialists across 12 transverses of the 24-h slides. A correction factor was applied to give the pollen count for each family for that 24-h period. Resulting count figures were converted into pollen grains per cubic meter of air and classified as the previous day’s exposure measure. Count data were available for family Poaceae (all grass combined) and the following tree genus: *Betula* (birch), *Fraxinus* (ash), *Platanus* (London Plane), *Quercus* (oak), and *Salix* (willow). Of note, ‘grass’ pollen is counted in total, (i.e. all genus and species in the family), as visual identification of species-specific pollen types is not possible (as opposed to pollen from different tree species). In addition, a count of total pollen concentration for the five types of tree was derived, and used as a further pollen predictor variable.

From the annual monitoring period, we focused on a common data analysis period for each year, from 1 April to 31 August 2005–2011 (153 days per year, total 1071 days). There was some variation in the annual monitoring periods, which at their extremes ran from 5 March (2008) to 21 September (2006), as well as missing data on both specific pollen types and on all pollen (109 days missing data, due to either mechanical failure of Burkard trap, early closure of monitoring station, and/or technician availability). The analysis period 1 April to 31 August covered the peak counts across all pollen types and had relatively limited missing data (although monitoring ended on 2 August 2009 and on 1 August 2011 due to the closure of the station). The samples of observation days used in estimation models of 0–7-day lagged exposure effects varied by pollen type (grasses, tree species). Calculation of lagged pollen exposure for the first week of April used monitoring station data from the final week of March, where available.

Due to the potential for measurement error in pollen counts (see “Discussion” section), pollen was modelled as both count data and as a categorical variable. Categorization used, where applicable, thresholds varying by pollen type that are used in the derivation of the ‘traffic light’ pollen alert warnings issued for the UK by the Met Office (with days classed as Low, Medium, High, or Very High pollen levels; http://www.metoffice.gov.uk/health/public/pollen-forecast, see TS6-11, supplementary material). The Met Office pollen warnings do not reference specific pollen types, but are derived from the analysis of counts against thresholds for the main allergenic pollen types at any one time throughout the forecast season based on significance in terms of greatest impact to the majority of sufferers. No threshold is available for willow pollen, and therefore, no categorical model was specified.

### Air pollution data

Data from government air pollution monitoring stations from the Automatic Urban and Rural Network (http://uk-air.defra.gov.uk/data/) were used to calculate the mean daily concentration (μg/m^3^) of air pollutants including particulate matter with aerodynamic diameter under 10 μm (PM_10_), nitrogen dioxide (NO_2_), sulphur dioxide (SO_2_), and ozone (O_3_). Data were obtained for urban and suburban background stations (see Fig. 1) that had <20% missing data from the periods 25 March to 31 August 2005–2011 (details presented in Table 1). Missing station values were imputed in a separate imputation model using chained equations in which complete data on the asthma outcome, day of year, year, humidity, precipitation, and temperature were included. The mean of 20 imputations for each station was used to fill missing station data. Following imputation, the daily mean concentration for each pollutant across London was calculated as the mean across all included stations.

**Table 1:** Mean daily air pollutant concentrations and proportion of data missing per station/pollutant, observation days 25 March to 31 August 2005–2011 (*n* = 799)

	Mean (SD), μg/m^3^				Missing data (% of values imputed)			
	PM_10_	NO_2_	SO_2_	O_3_	PM_10_	NO_2_	SO_2_	O_3_
Bexley, suburban background	–	27.49 (13.11)	4.37 (5.49)		–	4.0	7.3	–
Bloomsbury, urban background	23.34 (11.65)	50.02 (16.28)	3.25 (3.07)	34.90 (14.56)	6.9	8.8	7.1	8.5
Eltham, suburban background	–	21.53 (11.97)	–	49.93 (16.35)	–	3.5	–	3.2
Haringey Priory Park South, urban background	–	–	–	50.53 (16.98)	–	–	–	9.4
Hillingdon, urban background	–	45.85 (22.11)	–	35.08 (18.56)	–	11.2	–	3.4
North Kensington, background urban	22.16 (10.05)	30.43 (14.26)	2.45 (2.62)	49.94 (17.07)	16.9	3.2	2.7	7.6
Teddington, urban background	–	–	–	58.60 (17.65)	–	–	–	3.8
Westminster, urban background	–	37.14 (16.86)	3.22 (3.05)	45.03 (15.90)	–	8.5	4.6	5.0

### Meteorological data

Data from the British Atmospheric Data Centre (http://badc.nerc.ac.uk) were used to calculate the mean of the minimum and maximum dry bulb temperatures (°C) for seven weather monitoring stations within Greater London, which reported on at least 75% of days. Missing station values were imputed in a separate imputation model, and these were combined into a single daily mean series using previously established methods (Armstrong et al. 2011). A new metric ‘recent temperature’, the index providing the best fit in exploratory analyses, was derived as the mean of this temperature on each day and the previous 3 days. Precipitation and relative humidity measures, obtained via MEDMI (https://www.data-mashup.org.uk) (Hajat et al. 2017), were derived as the mean of 53 and 12 weather stations, respectively, within 30 km of the city centre (here 51.49° N, 0.085° E). The daily mean value for each weather parameter was a simple mean across all stations (following imputation of missing values where necessary).

### Model specification

**Fig. 2:**
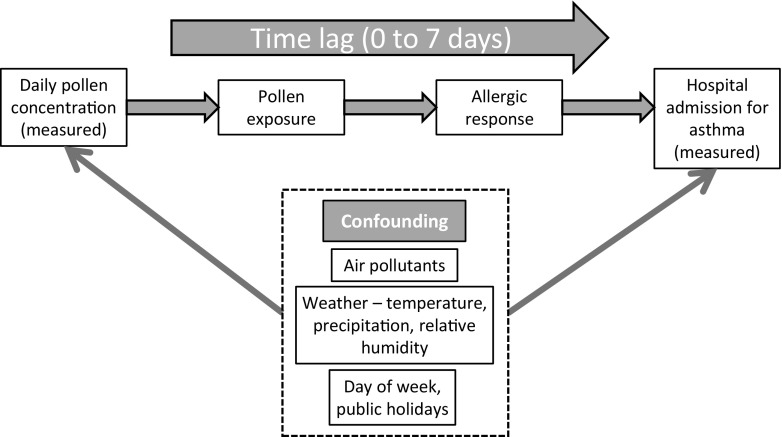
Conceptual framework for the exposure-outcome pathway, outlining the conceptual framework underpinning the analytical approach (model accounts for day of week (plus public holiday) effect)

Figure 2 depicts the conceptual framework underpinning the analyses. Model development followed the procedures recommended by Bhaskaran et al. (2013). Estimation of the impact of pollen types on asthma admissions in an ecological time series regression assumed a Poisson distribution with scale over-dispersion. We used natural cubic splines of time to flexibly model slow-changing seasonal patterns in the asthma data. Since these splines are parameterized, we used a generalized linear model rather than a generalized additive model, since the latter approach is only necessary when using non-parametric spline functions. In this case, spline functions were used to summarize the relationships in preliminary investigations of the functional form, and supported the assumption of the linear functional form. Preliminary investigations of the cross-correlation functions between the asthma outcome and explanatory variables informed the model development.

Within-season trend was accounted for using natural cubic splines of observation days in study seasons with five degrees of freedom per annual season (four internal knots placed equidistantly within each season), determined by examination of residual autocorrelation. Sensitivity analyses showed the results to be robust to a more aggressive control for within-season trend using eight degrees of freedom per annual season. Regression models were specified to estimate the change in risk of asthma hospitalization at the 95th percentile versus the 0th percentile associated with 0–7-day lags in unconstrained distributed lag models. We took this approach to reporting associations since it permits direct comparison of effect sizes between pollen types, and was comparable to the approach used in a recent study to examine the relationship between tree pollen counts and hospital admissions in New York City (Ito et al. 2015). All analyses were carried out with the ‘glm’ command in Stata 13 (StataCorp, College Station, USA).

Models further adjusted for 0–7-day lags of PM_10_, NO_2_, SO_2_, and O_3_ with an unconstrained lag form and a linear functional form specified, and for 0–7-day lags of natural cubic splines of recent temperature (as defined above), precipitation, and relative humidity with an unconstrained lag form and with two degrees of freedom for the functional form. Models also included categorical variables for day of the week and public holidays (since outdoor activity, air pollution levels, and hospital attendance may vary accordingly), and to adjust for any secular trends, a linear term for time was included. Residual autocorrelation from this initial specification was further adjusted for in the final specification by the inclusion of 1-day-lagged deviance residual terms.

## Results

The total number of adult (age 16–64 years) asthma admissions included across the entire study period was 11,984 (although the number considered in each regression model varied according to the subset of days included). Overall, the models demonstrated an association between asthma hospitalizations and grass pollen concentrations, but not tree pollen, after controlling for air pollution and weather variables. Descriptive statistics on the daily variables included in the estimation samples for the grass pollen models are presented in Table 2. These indicate, for example, that across 799 observed days included in the grass pollen models, there were a mean of 11.3 emergency admissions for asthma per day, with a minimum of 2 and a maximum 89. Across the same set of observed days, the mean grass pollen count was 24.5 grains/m^3^, with a range from 0 to 474 grains/m^3^. Equivalent descriptive statistics for the tree pollen model estimation samples are provided in supplementary tables for brevity (TS1-5).

**Table 2:** Descriptive data on the daily variables: grass pollen model (*n* observation days = 799)

Variable	Mean	SD	p10	p25	p50	p75	p90	Min	Max
Asthma admissions	11.26	5.42	6.00	8.00	11.00	13.00	16.00	2	89
Grass pollen count (grains/m^3^)	24.51	46.46	0.00	1.00	6.00	26.00	69.00	0	474
Mean daily O_3_ (μg/m^3^)	46.31	14.72	28.41	35.71	45.12	54.86	64.43	7.43	107.29
Mean daily PM_10_ (μg/m^3^)	22.72	10.21	12.71	15.50	20.50	27.77	35.50	7.50	86.00
Mean daily SO_2_ (μg/m^3^)	3.39	3.33	1.25	1.75	2.50	3.50	6.50	0.00	26.48
Mean daily NO_2_ (μg/m^3^)	35.53	12.77	22.00	26.00	32.76	42.75	52.67	12.00	91.50
Mean daily temperature (°C), over 0 to 3 day lag	15.40	3.48	10.57	12.97	15.87	17.55	19.33	4.49	24.96
Daily precipitation (0.1 mm)	1.59	3.09	0.00	0.00	0.20	1.87	4.89	0.00	28.82
Daily mean humidity (%)	69.41	9.93	57.56	61.88	68.29	76.01	83.30	36.83	97.04

*p10* 10th percentile, *p90* 90th percentile

**Fig. 3:**
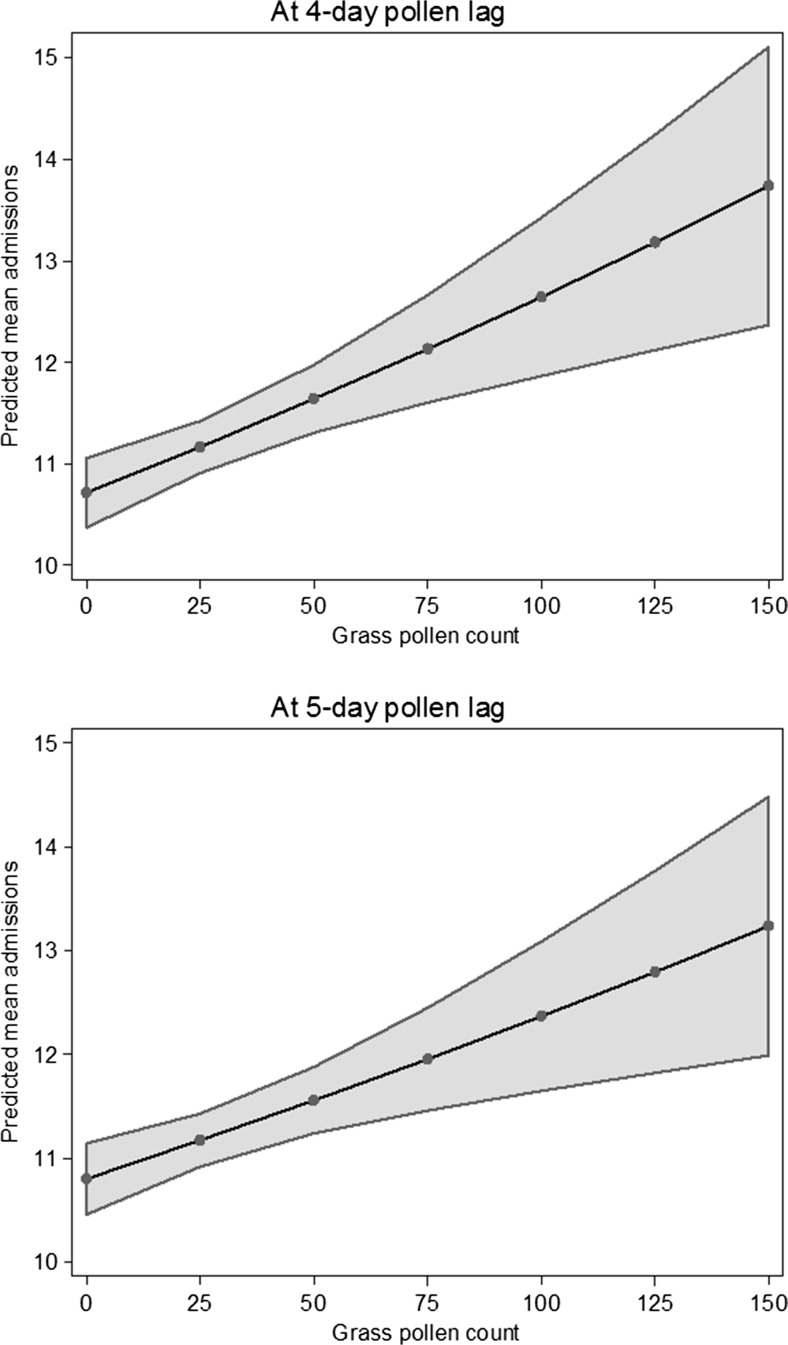
Predicted asthma admissions (95% CI) at representative grass pollen counts at 4-day and 5-day lags

Effect estimates at 0–7-day lags from the time series regression models are presented in Table 3, as the percentage change in the count of emergency in-patient admissions for asthma associated with 0 to 95th percentile increases in pollen (i.e. total grasses, the five tree types, and the total of these tree pollen concentrations). The number of days in which the 95th percentile count of pollen was observed or exceeded is tabulated by year in Table 4: for all pollen types, pollen counts reached the 95th percentile in at least 6 of the 7 years, and on several days in most years. Results in Table 3 indicate that higher grass pollen counts were associated with increased admissions at 4- and 5-day lags (*p* < 0.001); predicted admissions against grass pollen counts of 0–150 are presented in Fig. 3. In contrast, there was little observed effect from any of the tree pollen, considered alone or in total. Increases in admission rates were associated with ash pollen at a 4-day lag (*p* = 0.044) and willow pollen at a 2-day lag (*p* = 0.025), but without clear patterns or a priori specification of these lags, and because of the multiple comparisons being performed, it is difficult to infer causal association. These findings are unlikely to be the result of residual confounding with daily background air pollutant levels, since the correlations between air pollutants and species pollen were generally low (Table 5).

**Table 3:** Percentage change (with 95% confidence interval) in emergency asthma admissions associated with 0–95th percentile increase^a^ in pollen exposure

	Grass	Birch	Ash	London Plane	Oak	Willow	All trees combined
No. of obs.	799	737	686	738	716	694	678
0-day lag	−6.18 (−14.69, 2.33)	1.44 (−7.22, 10.1)	2.15 −(2.53, 6.84)	0.24 (−7.24, 7.7)	−5.3 (−14.61, 4.03)	−0.15 (−6.87, 6.61)	−4.01 (−17.95, 9.93)
1-day lag	−6.69 (−16.19, 2.82)	1.33 (−7.59, 10.26)	−0.18 (−5.66, 5.32)	−5.32 (−14.59, 3.96)	0.99 (−9.46, 11.44)	1.58 (−4.47, 7.66)	−8.65 (−24.08, 6.78)
2-day lag	6.2 (−3.28, 15.69)	2.77 (−6, 11.52)	2.32 (−2.1, 6.76)	0.1 (−9.67, 9.85)	7.46 (−2.55, 17.49)	6.96* (0.86, 13.1)	2.06 (−13.6, 17.74)
3-day lag	1.24 (−7.67, 10.15)	0.06 (−8.59, 8.7)	0.33 (−3.83, 4.5)	−4.92 (−15.19, 5.34)	−6.73 (−16.88, 3.43)	4.54 (−1.42, 10.54)	−10.82 (−26.86, 5.21)
4-day lag	17.23* (8.93, 25.54)	3.1 (−5.56, 11.78)	3.99* (0.1, 7.89)	5.27 (−4.38, 14.91)	−4.93 (−15.74, 5.89)	4.07 (−1.82, 9.98)	4.59 (−10.66, 19.87)
5-day lag	14.11* (6.22, 22.01)	−5.77 (−14.29, 2.75)	2.63 (−0.37, 5.62)	−6.19 (−15.75, 3.38)	2.92 (−7.12, 12.97)	−3.22 (−9.48, 3.08)	−9.45 (−24.66, 5.75)
6-day lag	5.95 (−2.04, 13.94)	1.76 (−6.36, 9.89)	0.66 (−2.24, 3.57)	0.47 (−9.07, 10.03)	0.6 (−9.69, 10.9)	5.29 (−0.47, 11.07)	0.89 (−14.32, 16.17)
7-day lag	3.31 (−4.21, 10.83)	−1.78 (−9.3, 5.73)	1.45 (−1.7, 4.6)	−1.19 (−8.9, 6.52)	6.51 (−2.93, 15.96)	1.78 (−3.9, 7.5)	2.67 (−11.66, 16.99)

Bold results indicate 95% confidence intervals excluding the null. All models adjusted for PM_10_, NO_2_, SO_2_, and O_3_; temperature; precipitation and relative humidity (lags as specified in text); day of week; and public holidays

*p < 0.05

^a^Zero to 95th percentile increases (grains/m^3^): grass = 104; birch = 195; ash = 18; London plane = 262; oak = 115; willow = 12

**Table 4:** Number of days at or exceeding the estimation sample 95th percentile pollen count^a^

	Grass	Birch	Ash	London Plane	Oak	Willow	All trees total combined
2005	8	2	2	7	4	1	3
2006	7	7	5	5	9	4	6
2007	6	2	2	6	2	4	6
2008	14	7	7	2	3	2	3
2009	5	4	2	2	9	3	6
2010	1	10	15	6	0	8	4
2011	0	6	3	9	9	14	6
Total	41	37	36	37	36	36	34

^a^Grass = 104; birch = 195; ash = 18; London plane = 262; oak = 115; willow = 12

**Table 5:** Correlation coefficients between pollen counts and air pollutant measures for estimation sample observations

	O_3_	PM_10_	SO_2_	NO_2_
Grass pollen count	0.21	0.07	0.09	−0.03
Birch pollen count	−0.01	0.20	0.14	0.29
Ash pollen count	0.01	0.06	0.09	0.18
London plane pollen count	0.00	0.29	0.13	0.26
Oak pollen count	0.13	0.41	0.09	0.31
Willow pollen count	0.09	0.19	0.07	0.21
All trees total	0.04	0.37	0.17	0.37

Descriptive statistics and results from regression of admissions against categorical pollen measures reflecting Met Office warning levels are presented in supplementary Tables TS6-11. Supplementary Table TS6 indicates the number of observation days for each of the five pollen types falling into the threshold categories used by the Met Office, and regression results are in TS7-11. The categorical model for grass pollen (TS7) produced similar results to the continuous model. ‘Very high’ grass pollen days were associated with increased incidence of asthma admissions relative to ‘low’ grass pollen days at 2–5-day lags, with a peak incidence rate ratio (IRR) of 1.46 (95% confidence interval [CI] 1.20–1.78) at a 3-day lag. ‘High’ versus low grass pollen days were only associated with increased admissions at a 3-day lag (IRR 1.14; 95% CI 1.01, 1.27). However, very high grass pollen days were also associated with a subsequent decrease in admission rate at 0–1-day lags (with the lowest IRR 0.72; 95% CI 0.59, 0.87 at 0 days).

Again, consistent with models fit with continuous pollen exposure variables, there were few clear associations between categorical tree pollen levels and asthma admissions. Selecting those results with a *p* value <0.05, there was a suggestion of an association with ash pollen (TS9) at a 2–3-day lag; peak IRRs comparing high (13 observation days) versus low (659 observation days) ash pollen at 2-day lag IRR = 1.52 (95% CI 1.01–2.27); and very high (3 observation days) versus low at 2-day lag IRR = 2.01 (95% CI 0.65–6.28). Categorical models also suggested higher admission rates at 5-day lag comparing ‘medium’ and low London plane pollen days (TS10), but also indicated *lower* admission rates at 4-day lag comparing very high to low oak pollen days (TS11). Whilst there were a small number of tree pollen category/lag day combinations giving rise to apparent associations, these had no clear pattern, and were subject to very wide confidence intervals due to the small numbers of days with higher levels of tree pollen.

## Discussion

### Key findings

Ecological time series analysis across 7 years found evidence of an association between daily total grass pollen concentration and adult emergency hospital in-patient admissions for asthma in London. There was a lag between exposure and admission of 4 to 5 days in models with grass pollen measured as a continuous variable, and 2 to 5 days where concentrations were categorized into Met Office pollen warning bands. There was no ‘same-day’ association apparent in the continuous models, although there was some suggestion in categorical models of a reduced risk of admission on the same day or the day after very high grass pollen counts (followed by increased risk at days 2–5). Associations observed in these models were apparent after adjustment for key potential confounders including air pollutant concentrations, weather, and day of week. There were no clear patterns of association between the number of admissions and concentrations of pollen from five tree types (birch, ash, London plane, oak, willow), either when considered individually or collectively. Whilst there were some suggestions of associations, there were very small numbers of observation days at higher levels (particularly important for categorical models), and the multiple comparisons and lack of a priori specific hypotheses mean that these isolated findings could be spurious.

The findings for grass pollen are consistent with previous evidence suggesting short-term increases in hospitalizations for asthma associated with pollen concentrations e.g. Erbas et al. (2007). The observed lag period is also consistent with some previous work; for example Tobías et al. (2004) found a lag of 3 days investigating total grass pollen levels and emergency asthma admissions in Madrid, Spain. However, the finding from the categorical models of a reduced risk of admission on the same day or day after very high grass pollen levels (relative to low days) is difficult to explain, even though this was followed by increased risk at days 2 to 5. Reduced risk on high pollen days could possibly be due to a reverse causation effect, because of increased use of antihistamines and other medications and better asthma management when pollen levels are very high, as patients have increased awareness with increased symptoms.

The lack of any clear association between tree pollen concentrations and asthma admissions is not consistent with some previous work, such as a study in New York City which did find associations between tree pollen (including birch, ash, London plane, and oak) and subsequent asthma emergency department visits (Ito et al. 2015). One explanation for this difference in findings could be differences in vegetation mix/climate and consequent pollen concentrations between the two locations; mean concentrations of ash, birch, and oak pollen were much higher in the New York study than London (although mean London plane pollen concentrations were higher in London). It is also possible that some of the inconsistencies with existing evidence could also be explained by residual confounding, or by potential errors introduced by design limitations, as discussed below.

### Strengths and limitations

The application of an ecological time series regression model to examine correlations between daily pollen and hospital admissions allows investigation of these acute relationships whilst accounting for a variety of potentially confounding environmental variables known to be related to both pollen levels and asthma admissions (e.g. temperature, precipitation, air pollution). Time series regression models are ideally suited to this type of examination of associations between acute, transient environmental exposures and short-term health outcomes (Bhaskaran et al. 2013). The models used here have the advantage of not only examining associations between exposures and outcomes whilst adjusting for potential or known confounders at a daily level but also modelling of short-term fluctuations in the presence of long-term or seasonal trends, as well as allowing for any time lag between exposure and effect. Furthermore, day of the week and public holidays are known to influence hospital attendance, and these have been accounted for in the current models.

The approach capitalizes on population-scale health data for a large city and hence a large study population, alongside several environmental time series data sets that have been collected consistently over the study period. We found only low levels of correlation between measures of air pollution and pollen levels, reducing the likelihood of confounding, comparable with previous London-based research (Anderson et al. 1998). By considering categorical versions of pollen concentration variables, we are able to relate the findings to the warnings produced to alert the public to high levels of pollen.

The study is subject to a number of design and data limitations. A key issue, common to much environmental epidemiology research, is that of environmental exposure assessment for pollen and potential confounders. Pollen was measured at a single site, and the measurements assumed to apply to the entire London population. Whilst pollen levels are likely to vary within the city, leading to exposure estimation errors, previous work comparing two English cities has suggested that pollen measurements up to 41 km apart are highly correlated, with near-perfect agreement between daily alert/forecast categories (Pashley et al. 2009). Since the pollen monitor producing the data for this study was located in central London, the maximum distance to the Government Office Region border defining the study population was only around 32 km. Similarly, the analyses assumed that the summary measures of air pollution concentrations and weather variables applied uniformly across the population of the city. London covers a large geographical area and is expected to have a series of microclimates across the area, and air pollution concentrations are known to vary on a fine spatial scale. Whilst this use of ecological exposure measurements leads to potential exposure misclassification on important confounders, again, the approach is common in this type of time series epidemiology (Bhaskaran et al. 2013; Katsouyanni et al. 1996). It is likely that the daily trends in relative change in pollen and covariate values in different parts of the city are broadly uniform, despite higher/lower baselines due to local circumstances. The measure of particulates available from London monitors was the concentration of PM_10_; ideally, we would have allowed for confounding by PM_2.5_, but daily data for the study time period were not available.

Whilst daily data for 7 years were available, the relatively short length of the pollen season led to a limited number of days that could be included in each model (maximum 799 for the grass pollen models). The authors of a recent methodological study, which informed our analyses, recommended that time series analyses (on air pollution effects) need thousands of observation days with an average of tens of events per day, for credible precision and power (Bhaskaran et al. 2013). In our study, we had only 799 days of pollen measures (grass models) and on average 11.3 hospitalizations for asthma per day (range 2 to 89). Limited data meant that the statistical power was somewhat restricted, reflected in wide confidence intervals around estimates. Over-dispersion (high variability in patient counts) may further reduce precision; in addition, measures of pollen were also skewed with a median grass pollen count at five grains per cubic metre (range 0 to 474).

Evidence from past studies both supports (Cuinica et al. 2014) and opposes (Garty et al. 1998) the hypothesis that air pollution or its individual constituents may act synergistically to increase the risk of asthma exacerbations leading to hospital admittance. However, limited data meant that we were unable to investigate interaction effects between pollen and air pollution exposures here; e.g. stratification by high/low days of air pollution was not possible. Future, longer-term studies that are able to include more sampling periods could potentially overcome these limitations.

A related limitation of this study is the lack of days on which there were high levels of tree pollen. Unlike the combined grass pollen, which typically has a much longer flowering season with more days of higher pollen concentrations, the individual tree pollen has peak times over short periods of 2–4 weeks with lower concentrations, again limiting the statistical power to estimate impacts on asthma admissions. Additionally, since tree pollen seasons can overlap significantly (e.g. ash and birch), time series analysis may not be able to determine species-specific associations. However, the release of pollen from some species is known to occur over a short time period, and may only reach high levels for a few days; with sufficient data covering a number of years, these associations could still potentially be detected. This study only included data on pollen sampled from 1 April each year. Some trees (such as hazel, yew, alder, elm, willow, and poplar) tend to have their peak pollen release in March, and hence, their times of high pollen exposure may have been missed. Birch, ash, London plane, and oak tend to have peak pollen release in April, and so these times were usually included in this study.

Currently, grass pollen data are counted in total without separation of individual species. The potential for individuals being allergic to some and not all species of grass does exist, considering the exquisite sensitivity of the IgE molecule to recognize proteins (Nony et al. 2015). Asthma exacerbations are not only caused by immunological pathways, but mechanical irritation from dust/pollen may also act as a trigger (Gold and Wright 2005). Potential differences in size may also play a role in pollen dispersal and how far into the lungs the particles can reach. With the current measurement method, grass pollen concentration exhibits a low arc over the pollen season, initiating in mid-May and ending towards the end of July to early August. This is a different-shaped curve from the tree pollen measured at the species level, which tended to be a sharper, higher peak of counts over a shorter time span, in the case of oak pollen over a 3-week period. This probably has an evolutionary basis, where species release pollen at a specific time (instigated by accumulated temperature, humidity/precipitation, and potentially photoperiod) when the stigma of the flowers of the species are ready to accept the pollen to ensure pollination and not become covered with pollen from other species, wasting potential reproduction possibilities. It is thought that grass pollen at the species level may well follow a similar trend of short high-peaking pollination periods as observed with the tree pollen, and work is currently underway to examine this (Skjoth et al. 2015; Kmenta et al. 2016). The flowering of grasses in Vienna have shown a rolling phenology over the season with various species of grass from early April (e.g. *Alopecurus pratensis*) through to the end of July (e.g. *Cynodon dactylon*), with a high peak for the number of pollen grains around mid-May and mid-June (coinciding with *Holcus lanatus* and *Lolium perenne* pollination periods, respectively) (Kmenta et al. 2016).

Finally, this study only considered London. Whilst this single-city approach is again comparable with other studies (e.g. Madrid (Tobías et al. 2004), New York City (Ito et al. 2015)), care should be taken in extrapolating these data to the entire UK population (or indeed extrapolation to other areas and populations). London is not representative of the UK, although the population included here is very large, ca. 8.5 of 65 million UK inhabitants. Not only does it include a younger, more ethnically diverse population, but also it is located in the SE of England where climate, landscape, urban effects, and grass species differ to elsewhere in the country.

## Conclusions

In the population of London, UK, daily total grass pollen concentrations from 2005 to 2011 were associated with increased emergency hospital admissions for asthma amongst adults aged 16–64, with a lag of 2 to 5 days between exposure and admission. There was no substantive evidence of a similar association between asthma admissions and ash, oak, London plane, willow, or birch tree pollen concentrations, or the total concentration of pollen from all five trees. Future work would benefit from longer time series of pollen data, from measures of exposure to grass species-specific pollen, from ability to examine linkage between pollen and health outcomes at the individual level on high and low air pollution days, and potentially from multi-city studies.

## Electronic supplementary material

ESM 1 (PDF 482 kb)

## Abbreviations

ISAAC: International Study of Asthma and Allergies in Childhood
HES: Hospital Episode Statistics
ICD: International Classification of Disease
