# Supplementary material for: Pollen exposure and hospitalization due to asthma exacerbations: daily time series in a European city
Source: Int J Biometeorol. 2017 May 12;61(10):1837–48. doi: 10.1007/s00484-017-1369-2 (PMC5643363; doi:10.1007/s00484-017-1369-2)
Supplement: Supplementary file 1 — (PDF 482 kb) [file 484_2017_1369_MOESM1_ESM.pdf]

Pollen exposure and hospitalization due to asthma exacerbations: daily time series in a European city

International Journal of Biometeorology

Electronic Supplementary Material

Nicholas J Osborne[1,2], Ian Alcock[1], Benedict W Wheeler[1], Shakoor Hajat[3], Christophe Sarran[4], Yolanda Clewlow[4], Rachel N McInnes[1,4], Deborah Hemming[4], Mathew White[1], Sotiris Vardoulakis[1,3,5], Lora E Fleming[1]

Institutions

[1]European Centre for Environment and Human Health, University of Exeter Medical School, Truro, Cornwall, UK
[22]School of Public Health and Community Medicine, University of New South Wales, NSW 2052, Australia
[3]London School of Hygiene and Tropical Medicine, London UK
[4]Met Office Hadley Centre, Fitzroy Road, Exeter, EX1 3PB, UK
[5]Environmental Change Department, Centre for Radiation, Chemical and Environmental Hazards, Public Health England, Chilton, Oxon, OX11 0RQ, UK

Corresponding Author

Dr Nicholas J. Osborne, School of Public Health and Community Medicine, University of New South Wales, NSW 2052, Australia. phone: +61 431854846 fax: +61 (2) 9313 6185 email: n.osborne@unsw.edu.au

Tables TS1-5: Descriptive statistics for daily tree pollen model variables (cf. main paper Table 2).

TS1 Descriptive data on the daily variables: Birch pollen model (n. observation days = 737)

variable	mean	SD	p10	p25	p50	p75	p90	min	max
Asthma admissions	11.35	5.53	7.00	8.00	11.00	13.00	16.00	2	89
Birch pollen count	28.94	96.34	0.00	0.00	0.00	3.00	66.00	0	786
Mean daily O_3 µg/m^3	46.62	14.74	28.68	36.29	45.29	55.00	64.71	11.71	107.29
Mean daily PM_{10} µg/m^3	22.79	10.14	12.79	15.59	20.50	27.65	35.50	7.50	86.00
Mean daily SO_2 µg/m^3	3.40	3.39	1.25	1.75	2.42	3.50	6.50	0.00	26.48
Mean daily NO_2 µg/m^3	35.57	12.81	22.17	26.04	32.81	42.52	53.17	12.00	91.50
Mean daily temperature °C, over 0 to 3 day lag	15.42	3.40	10.82	13.13	15.84	17.50	19.22	4.49	24.96
Daily precipitation (0.1 mm)	1.61	3.14	0.00	0.00	0.19	1.81	5.03	0.00	28.82
Daily mean humidity (%)	69.43	10.13	57.45	61.82	68.14	76.20	83.44	36.83	97.04

TS2 Descriptive data on the daily variables: Ash pollen model (n. observation days = 686)

variable	mean	SD	p10	p25	p50	p75	p90	min	max
Asthma admissions	11.29	5.62	7.00	8.00	11.00	13.00	16.00	2	89
Ash pollen count	5.22	24.87	0.00	0.00	0.00	1.00	8.00	0	332
Mean daily O_3 µg/m^3	46.56	14.93	28.57	35.86	45.14	55.00	65.29	11.71	107.29
Mean daily PM_{10} µg/m^3	23.19	10.32	12.87	16.00	21.00	28.43	36.00	7.50	86.00
Mean daily SO_2 µg/m^3	3.46	3.42	1.25	1.75	2.50	3.50	6.75	0.00	26.48
Mean daily NO_2 µg/m^3	36.07	12.91	22.39	26.33	33.33	43.00	53.43	14.00	91.50
Mean daily temperature °C, over 0 to 3 day lag	15.36	3.50	10.65	12.92	15.72	17.53	19.38	4.49	24.96
Daily precipitation (0.1 mm)	1.63	3.15	0.00	0.00	0.20	1.92	4.89	0.00	28.82
Daily mean humidity (%)	69.79	10.15	57.58	62.44	68.50	76.67	83.71	36.83	97.04

TS3 Descriptive data on the daily variables: London Plane pollen model (n. observation days = 738)

variable	mean	SD	p10	p25	p50	p75	p90	min	max
Asthma admissions	11.35	5.53	7.00	8.00	11.00	13.00	16.00	2	89
London Plane count	45.52	169.20	0.00	0.00	0.00	9.00	101.00	0	2221
Mean daily O_3 µg/m^3	46.62	14.73	28.68	36.29	45.29	55.00	64.71	11.71	107.29
Mean daily PM_{10} µg/m^3	22.78	10.14	12.79	15.55	20.50	27.65	35.50	7.50	86.00
Mean daily SO_2 µg/m^3	3.40	3.39	1.25	1.75	2.41	3.50	6.50	0.00	26.48
Mean daily NO_2 µg/m^3	35.56	12.81	22.17	26.04	32.78	42.52	53.17	12.00	91.50
Mean daily temperature °C , over 0 to 3 day lag	15.42	3.40	10.82	13.13	15.84	17.50	19.22	4.49	24.96
Daily precipitation (0.1 mm)	1.61	3.14	0.00	0.00	0.20	1.81	5.03	0.00	28.82
Daily mean humidity (%)	69.42	10.12	57.45	61.82	68.11	76.20	83.44	36.83	97.04

TS4 Descriptive data on the daily variables: Oak pollen model (n. observation days = 716)

variable	mean	SD	p10	p25	p50	p75	p90	min	max
Asthma admissions	11.37	5.58	7.00	8.00	11.00	13.00	16.00	2	89
Oak pollen count	21.54	69.08	0.00	0.00	1.00	8.00	52.00	0	785
Mean daily O_3 µg/m^3	46.75	14.80	28.71	36.42	45.62	55.61	64.71	11.71	107.29
Mean daily PM_{10} µg/m^3	22.99	10.18	13.00	16.00	20.50	28.00	35.50	7.50	86.00
Mean daily SO_2 µg/m^3	3.41	3.41	1.25	1.75	2.44	3.50	6.57	0.00	26.48
Mean daily NO_2 µg/m^3	35.61	12.78	22.17	26.07	32.83	42.59	52.82	12.00	91.50
Mean daily temperature °C, over 0 to 3 day lag	15.49	3.32	10.94	13.15	15.84	17.51	19.25	7.07	24.96
Daily precipitation (0.1 mm)	1.64	3.18	0.00	0.00	0.20	1.83	5.03	0.00	28.82
Daily mean humidity (%)	69.57	10.19	57.45	61.84	68.27	76.57	83.63	36.83	97.04

TS5 Descriptive data on the daily variables: Willow pollen model (n. observation days = 694)

variable	mean	SD	p10	p25	p50	p75	p90	min	max
Asthma admissions	11.31	5.61	7.00	8.00	11.00	13.00	16.00	2	89
Willow pollen count	2.22	7.08	0.00	0.00	0.00	1.00	6.00	0	85
Mean daily O_3 µg/m^3	46.64	14.89	28.68	36.02	45.29	55.50	65.14	11.71	107.29
Mean daily PM_{10} µg/m^3	23.11	10.29	12.88	16.00	20.73	28.00	36.00	7.50	86.00
Mean daily SO_2 µg/m^3	3.44	3.41	1.25	1.75	2.50	3.50	6.75	0.00	26.48
Mean daily NO_2 µg/m^3	35.97	12.89	22.33	26.17	33.12	43.00	53.34	14.00	91.50
Mean daily temperature °C, over 0 to 3 day lag	15.35	3.49	10.69	12.92	15.69	17.51	19.33	4.49	24.96
Daily precipitation (0.1 mm)	1.61	3.14	0.00	0.00	0.20	1.87	4.84	0.00	28.82
Daily mean humidity (%)	69.67	10.16	57.45	62.36	68.36	76.65	83.63	36.83	97.04

TS6-11. Descriptive statistics and results from categorical models, classifying daily pollen counts to pollen alert levels issued for the UK by the Met Office, as described in the main text. These classifications are as follows:

- Grass pollen categories: **L**ow (reference) >=0 & <=29; **M**edium >=30 & <=49; **H**igh >=50 & <=149; **V**ery **H**igh >=150
- Birch pollen categories: L (reference) >=0 & <=39; M >=40 & <=79; H >=80 & <=199; VH >=200
- Ash pollen categories: L (reference) >=0 & <=29; M >=30 & <=49; H >=50 & <=199; VH >=200
- London Plane pollen categories: L (reference) >=0 & <=29; M >=30 & <=49; H >=50 & <=199; VH >=200
- Oak pollen categories: L (reference) >=0 & <=29; M >=30 & <=49; H >=50 & <=199; VH >=200
- (There are no Met Office thresholds for willow pollen)

Regression results in TS7-11 are adjusted for all potential confounders as per continuous models presented in the main text.

TS6. Number (%) of observed days in estimation samples across Met Office pollen warning categories

**Pollen count categories:** **Low Medium** **High** **Very High** **(grains/m³)**	**Grass**		**Birch**		**Ash**		**London Plane**		**Oak**	
	n	%	n	%	n	%	n	%	n	%
L 0-29	616	77.1								
M 30-49	59	7.4								
H 50-149	105	13.1								
VH 150+	19	2.4								
L 0-39			646	87.7						
M 40-79			23	3.1						
H 80-199			32	4.3						
VH 200+			36	4.9						
L 0-29					659	96.1	614	83.2	621	86.7
M 30-49					11	1.6	22	3	22	3.1
H 50-199					13	1.9	52	7.1	47	6.6
VH 200+					3	0.4	50	6.8	26	3.6

TS7. Incidence rate ratios for emergency asthma admissions associated with warning categories of grass pollen count (with reference to category 'Low')

	**Grass pollen categories model, n=799. Incidence rate ratios**		
	Medium >=30 & <=49	High >=50 & <=149	Very High >=150
0-day lag	0.95 (0.85-1.06)	0.98 (0.88- 1.09)	0.72* (0.59-0.87)
1-day lag	0.99 (0.88- 1.10)	0.96 (0.86- 1.08)	0.79* (0.65-0.97)
2-day lag	0.93 (.82 - 1.04)	0.94 (0.84-1.06)	1.42* (1.17-1.7)
3-day lag	1.02 (0.90- 1.14)	1.14* (1.01-1.28)	1.46* (1.20-1.78)
4-day lag	0.90 (0.80 1.01)	0.96 (0.86- 1.08)	1.30* (1.8-1.59)
5-day lag	0.95 (0.83- 1.07)	0.98 (0.88-1.10)	1.28* (1.07-1.55)
6-day lag	0.91 (0.80- 1.03)	0.94 (0.84- 1.06)	1.13 (0.93-1.36)
7-day lag	0.88 (0.78-1.00)	0.97 (0.86- 1.08)	0.95 (0.79-1.14)

* $p<0.05$

TS8. Incidence rate ratios for emergency asthma admissions associated with warning categories of birch pollen count (with reference to category 'Low')

	**Birch pollen categories model, n=737. Incidence rate ratios**		
	Medium >=40 & <=79	High >=80 & <=199	Very High >=200
0-day lag	0.97 (0.78-1.21)	1.02 (0.81-1.29)	1.07 (0.85-1.36)
1-day lag	0.98 (0.79-1.2)	0.94 (0.75-1.18)	0.99 (0.78-1.27)
2-day lag	0.98 (0.79-1.21)	1.03 (0.83-1.28)	1.0 (0.78-1.27)
3-day lag	1.08 (0.88-1.33)	1.03 (0.83-1.28)	1.02 (0.81-1.28)
4-day lag	0.86 (0.69-1.06)	0.98 (0.79- 1.21)	0.94 (0.74-1.19)
5-day lag	0.92 (0.75-1.13)	0.99 (0.80-1.22)	0.87 (0.69-1.10)
6-day lag	0.87(0.70-1.08)	1.19 (0.97-1.47)	1.08 (0.87-1.35)
7-day lag	0.99 (0.80-1.21)	1.05 (0.86-1.28)	0.95 (0.77-1.18)

* p<0.05

TS9. Incidence rate ratios for emergency asthma admissions associated with warning categories of ash pollen count (with reference to category 'Low')

	**Ash pollen categories model, n=686. Incidence rate ratios**		
	Medium >=30 & <=49	High >=50 & <=199	Very High >=200
0-day lag	0.90 (0.66- 1.24)	1.19 (0.78-1.82)	1.50 (0.62- 3.60)
1-day lag	1.13 (0.84-1.51)	1.09 (0.72-1.66)	0.80(0.25-2.57)
2-day lag	1.05 (0.79-1.40)	1.52* (1.01-2.27)	2.01 (0.65-6.28)
3-day lag	1.12 (0.86-1.46)	1.51* (1.04-2.19)	1.56 (0.60- 4.04)
4-day lag	0.89 (0.69-1.15)	1.25 (0.87-1.80)	1.60 (0.75- 3.37)
5-day lag	0.95 (0.75-1.21)	0.99 (0.70-1.38)	1.25 (0.69- 2.26)
6-day lag	0.88 (0.70-1.11)	1.10 (0.82-1.46)	0.98 (0.59-1.61)
7-day lag	1.02 (0.81-1.29)	1.17 (0.89-1.55)	1.59 (0.97-2.60)

*p<0.05

TS10. Incidence rate ratios for emergency asthma admissions associated with warning categories of grass pollen count (with reference to category ‘Low’)

	**London Plane pollen categories model, n=738. Incidence rate ratios**		
	Medium >=30 & <=49	High >=50 & <=199	Very High >=200
0-day lag	1.07 (0.87-1.31)	1.01 (0.84-1.21)	0.98 (0.78- 1.24)
1-day lag	1.15 (0.93-1.42)	1.15 (0.94-1.40)	1.00 (0.78-1.29)
2-day lag	1.00 (0.80-1.24)	1.02 (0.83-1.25)	1.00 (0.77-1.29)
3-day lag	0.95 (0.77-1.17)	1.01 (0.82- 1.24)	0.96 (0.74-1.24)
4-day lag	0.98 (0.80- 1.20)	0.95 (0.78-1.17)	0.92 (0.70-1.19)
5-day lag	1.23* (1.00-1.49)	1.00 (0.81-1.24)	1.00 (0.76-1.32)
6-day lag	1.00 (0.81-1.23)	1.02 (0.83-1.26)	0.95 (0.72-1.26)
7-day lag	1.09 (0.89-1.33)	0.95 (0.79-1.16)	0.96 (0.74-1.23)

* p<0.05

TS11. Incidence rate ratios for emergency asthma admissions associated with warning categories of oak pollen count (with reference to category ‘Low’)

	**Oak pollen categories model, n=716. Incidence rate ratios**		
	Medium >=30 & <=49	High >=50 & <=199	Very High >=200
0-day lag	0.95 (0.78-1.16)	1.00 (0.82- 1.21)	0.80 (0.60-1.06)
1-day lag	0.82 (0.66-1.02)	1.04 (0.84- 1.28)	1.01 (0.74- 1.37)
2-day lag	1.15 (0.93-1.43)	1.13 (0.91- 1.40)	1.18 (0.86- 1.62)
3-day lag	1.02 (0.83-1.26)	0.99 (0.80- 1.23)	0.95 (0.69 - 1.31)
4-day lag	1.07 (0.86- 1.32)	0.85 (0.69- 1.04)	0.69* (0.49-0.96)
5-day lag	1.14 (0.91-1.42)	1.14 (.93- 1.40)	1.21 (0.88- 1.68)
6-day lag	1.16 (0.94-1.43)	0.99 (0.81 - 1.22)	1.15 (0.83-1.59)
7-day lag	1.02 (0.83-1.27)	1.07 (0.89 - 1.28)	1.17 (0.87-1.59)

* $p<0.05$
